# Study protocol for a pilot high-intensity interval training intervention in inpatient mental health settings: a two-part study using a randomised controlled trial and naturalistic study design

**DOI:** 10.1186/s40814-021-00937-6

**Published:** 2021-11-08

**Authors:** Rebecca Martland, Juliana Onwumere, Brendon Stubbs, Fiona Gaughran

**Affiliations:** 1 Department of Psychosis Studies, Institute of Psychiatry, Psychology and Neuroscience (IoPPN), King’s College London, London, UK; 2 South London and Maudsley NHS Foundation Trust, London, UK; 3 Department of Psychology, Institute of Psychiatry, Psychology and Neuroscience (IoPPN), King’s College London, London, UK; 4 Department of Psychological Medicine, Institute of Psychiatry, Psychology and Neuroscience (IoPPN), King’s College London, London, UK

**Keywords:** Severe mental illness, Physical health, Exercise, Intervention, Inpatient

## Abstract

**Background:**

Severe mental illnesses (SMI), including schizophrenia spectrum disorder, bipolar disorder and major depressive disorder, are associated with physical health comorbidities and premature mortality. Physical activity and structured exercise have a beneficial impact on cardiometabolic risk and ameliorate mental health symptomology and cognition. This protocol describes a feasibility study for a high-intensity interval training (HIIT) intervention among inpatients with SMI, to improve their physical and mental health.

**Methods:**

The feasibility study follows a two-part design owing to COVID-19-related adaptations to project design: (a) a non-blinded randomised controlled trial (RCT) of 12 weeks of bicycle-based HIIT, delivered twice weekly in a face-to-face, one-to-one setting, compared to treatment as usual (TAU) and (b) a naturalistic study of inpatient HIIT; eligible participants will be invited to two sessions of HIIT per week, delivered by the research team remotely or in person. Additionally, participants in the naturalistic study may use the bike to conduct self-directed sessions of their chosen length and intensity. We will measure the feasibility and acceptability of the HIIT intervention as primary outcomes, alongside secondary and tertiary outcomes evaluating the physical, mental and cognitive effects of HIIT. The study aims to recruit 40 patients to the RCT and 6–8 patients to the naturalistic design.

**Discussion:**

Exercise is a modifiable lifestyle barrier that can reverse cardiometabolic disease risk. If HIIT is found to be feasible and acceptable in inpatients with SMI, there would be scope for large-scale work to evaluate the clinical, cost and implementation effectiveness of HIIT in inpatient mental health settings.

**Trial registration:**

ClinicalTrials.gov NCT03959735. Registered June 22, 2019.

**Supplementary Information:**

The online version contains supplementary material available at 10.1186/s40814-021-00937-6.

## Background

Severe mental illnesses (SMI), including schizophrenia spectrum disorder, bipolar disorder and major depressive disorder (MDD), are the leading causes of years lived with disability, affecting approximately 3–5% of the population [1–3]. Along with the impact of the mental health symptoms and reduced daily functioning, SMIs are associated with physical health comorbidity and an associated premature mortality amounting to 13–20 years [4, 5]. Cardiometabolic diseases, including the metabolic syndrome, diabetes mellitus, obesity, and coronary heart disease, are two-fold higher in people with SMI [6, 7]. In addition, people with SMI typically experience cognitive impairment, socio-occupational difficulties and reduced quality of life (QoL) which may worsen over time [8–11].

The newly published WHO guidelines report that frequent physical activity and structured exercise have a beneficial impact on cardiometabolic and cardiovascular disease risk, and ameliorate symptoms and QoL for those living with these complications [12]. Further, regular exercise can improve mental health, cognition and sleep quality, in both the general population and those with severe mental health conditions [13–16] and may aid smoking cessation in the long term [17, 18]. Acute exercise bouts can also have immediate effects on appetite and cigarette cravings [19–21], cognitive functioning [22] and positive wellbeing in healthy and clinical populations [22–24]. This said, people with SMI engage in significantly less moderate and vigorous-intensity exercise than the general population and are less likely to meet physical activity guidelines of 150 min of exercise per week [25]. Thus, low levels of physical activity are recognised as contributing towards physical, mental and cognitive ill health in this population group [13], along with higher levels of tobacco consumption and antipsychotic-induced weight gain. Physical activity is a modifiable lifestyle risk factor; thus, a focus of current research is to increase levels of physical activity in people with SMI and to promote uptake of structured exercise interventions [26]. For example, the recent European Psychiatric Association (EPA) guidance draws on 20 past systematic reviews in the SMI population and suggests physical activity should be used as an adjunctive treatment in SMI to improve mental health symptoms, physical health, cognition and quality of life [13].

High-intensity interval training (HIIT) is a type of exercise characterised by alternating short bursts (typically 30 s to 4 min) of high-intensity exercise interspersed by similar length periods of light exercise or rest, repeated for typically 10–25 min [27]. This popular fitness approach yields positive effects on physical and mental health in the general population and may be more beneficial in terms of improvements in cardiometabolic health when compared to traditional moderate-intensity continuous training (MICT) approaches [28]. Preliminary work has sought to establish the effectiveness of HIIT in people with schizophrenia spectrum disorders [29–35], major depressive disorders [36–39] and adults with self-reported psychiatric problems [40] in interventions of duration 12 days to 6 months. This work has been summarised in two recent meta-analyses, whereby significant improvements in depressive symptoms and cardiorespiratory fitness were reported post HIIT intervention, and there was a suggestion that HIIT may be more beneficial in terms of improvements in depressive symptoms when compared to MICT [41, 42]. Further, HIIT may improve cognitive measures including verbal learning and overall neurocognition [35]. This said, there is inconsistency concerning the effects of HIIT on psychopathology, social and global functioning and anthropometric measures, which is hampered by small sample sizes and a paucity of clinical trials. The total number of participants with an SMI allocated to HIIT ranges from 8 [40] to 43 [29, 35] across trials, and meta-analysis data summarised findings from just 366 participants with SMI, including comparison groups. To add, previous work in people with SMI has reported a low rate of adverse events (AE) including no acute injuries or cardiovascular events, completion rates of approximately 71% and a mean attendance at sessions of 74%, suggesting good feasibility and acceptability, although these measures have only been reported in roughly half of HIIT trials in people with SMI to date [41, 42].

To add, two trials have sought to establish the acute effects of HIIT in those with a mental illness [43, 44]. In patients with schizophrenia and depression, there was an improvement in positive affect and wellbeing, and a reduction in psychological distress and state anxiety from pre-training to 15 min post-HIIT [43]. In adolescents hospitalised with MDD, suicidal ideation, stress and anxiety disorders, there was a suggestion that acute bouts of HIIT may improve inhibitory control for up to 30 min post-exercise [44].

Despite the initial evidential support for HIIT in people with SMI, little work has been undertaken with patients receiving treatment on psychiatric wards, where embedding new ways of working can often be associated with logistical challenges specific to the setting. For example, shift work for staff, access and space issues, and the profile of needs and acuity in the patient group. Inpatients are typically more acutely unwell and receive higher doses of psychotropic medication [45] and polypharmacy [46]. These factors are likely to impact service implementation and patient engagement with an exercise intervention, which in turn will impact their efficacy. To date, four papers have assessed the effect of HIIT in inpatients with MDD [36–39], only one has assessed HIIT in inpatients with schizophrenia [32] and another has looked at the acute effects of a single bout of HIIT in adolescents receiving inpatient mental health treatment [44], highlighting a need for further research in this population and setting. Moreover, a recent qualitative analysis explored perspectives on implementing HIIT interventions in inpatient mental health settings [47]. Across seven focus groups, in inpatients with SMI, carer and staff groups, HIIT was seen positively, with beliefs that it would help inpatients feel more relaxed, build their fitness, and provide a break from the monotony of ward environments. This said, concerns were noted related to patient motivation, safety, especially for those with chronic physical health comorbidities, and practical logistical factors, including having access to the right sports clothing and staff availability to supervise [47].

### Aims

The primary aim of this feasibility study therefore is to determine whether HIIT is acceptable and feasible to implement in a psychiatric inpatient setting with patients with a broad range of severe mental illnesses, while the secondary aim is to investigate if the HIIT intervention improves mental health symptoms, including psychiatric symptoms, depression, anxiety, stress, sleep and mental wellbeing; cognition; and along with physical activity measures including increases in weekly physical activity, motivation to engage in exercise and anthropometric measures. The tertiary aim is to determine whether a single bout of HIIT leads to acute changes in psychological states and appetite and cigarette cravings. Conclusions drawn from our secondary and tertiary aims will offer an indication of the therapeutic benefits of HIIT in preparation for larger scale work.

## Methods/design

### Design

This pilot study follows a two-part design as a result of COVID-19 and consequent social restrictions.

- A parallel non-blinded randomised controlled trial (RCT) of HIIT compared to treatment as usual (TAU). Recruitment commenced in January 2020 and was suspended in March 2020 in accordance with COVID-19 social restrictions and corresponding Health Research Authority (HRA) guidelines. Participants randomised to HIIT were allocated to 12 weeks of HIIT twice weekly, delivered in a face-to-face setting. Participants assigned to TAU were instructed to maintain their usual dietary habits, and no restrictions were applied to their current level of physical activity.
- The study design was then adapted to a COVID-19 compliant naturalistic study of HIIT uptake in inpatient psychiatric settings. All eligible participants will be invited to take part in two sessions of HIIT per week, delivered by the research team remotely or in person depending on COVID-19 social restrictions. Additionally, patients will be able to use the bike to conduct self-directed sessions of their own chosen length and intensity. No time limit for participation will be applied. Recruitment to the naturalistic study design began in May 2021 and is ongoing.

**Fig. 1:**
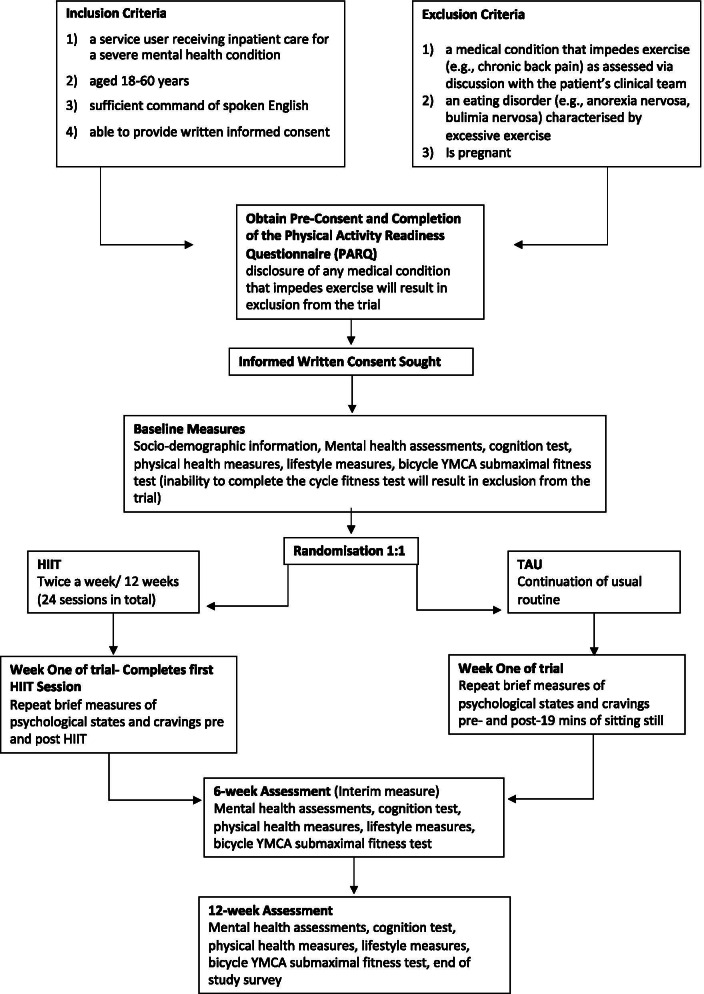
Study flow diagram Part A

**Fig. 2:**
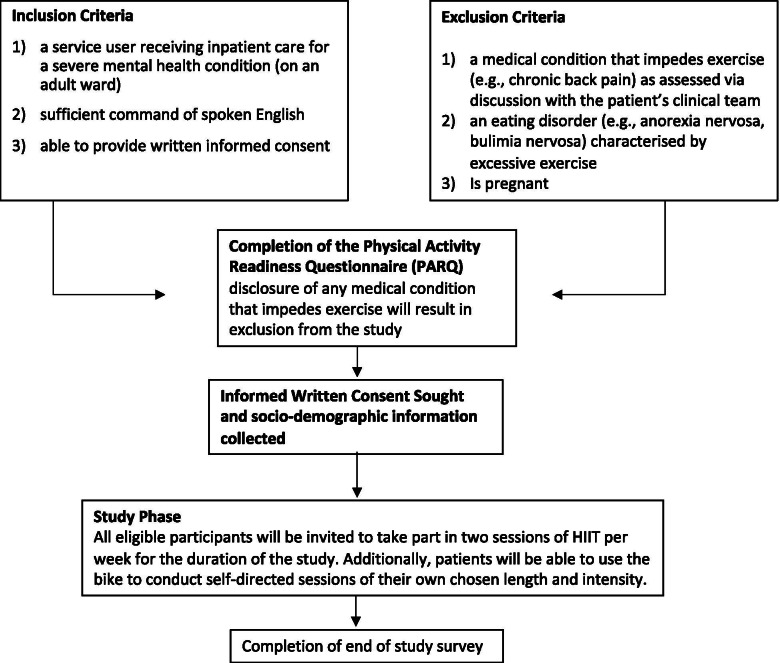
Study flow diagram Part B

**Fig. 3:**
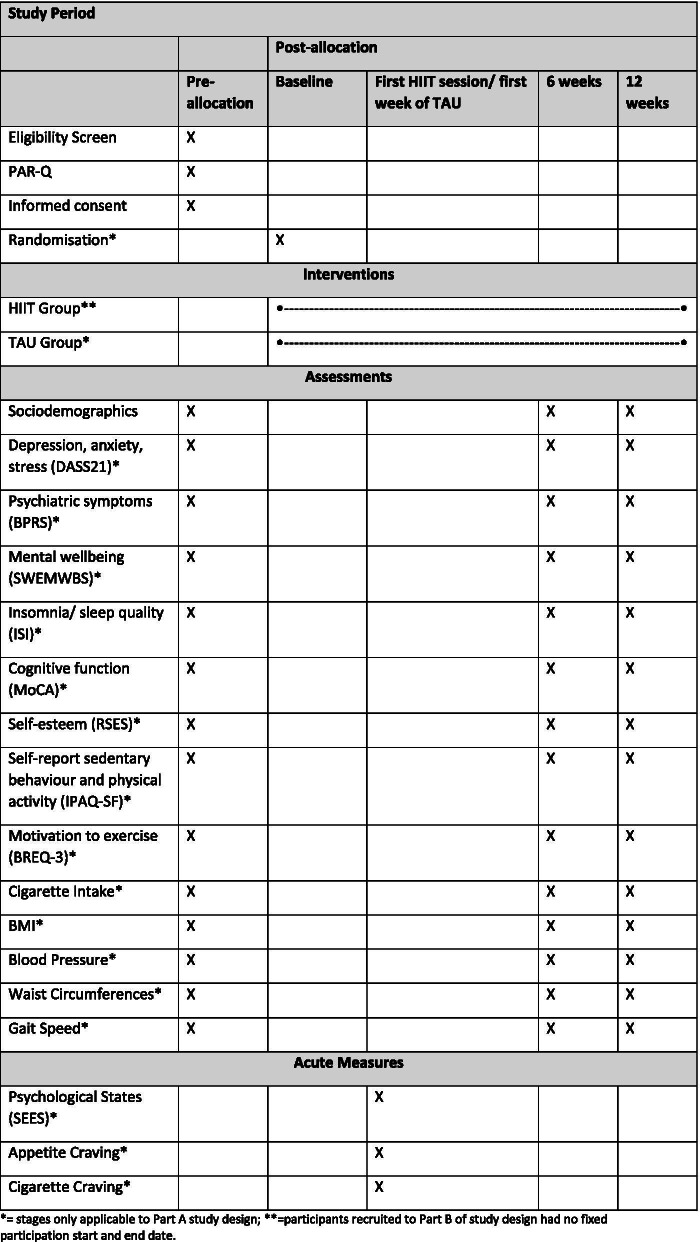
Schedule of enrolment, interventions and assessments in RCT

The novel two-part design is based on the need to adapt to COVID-19 restrictions. The pilot design (Part A) is registered online (ClinicalTrials.gov Identifier: NCT03959735) and has been reported in accordance with the CONSORT for reporting of pilot and feasibility trials [48], and the SPIRIT guideline for main trials was consulted [49]. Ethical approval was obtained from London Bloomsbury Ethics Committee (IRAS ID: 263996, REC reference 19/LO/0901) for part A of the research and governance approval was granted from local National Health Service (NHS) bodies for part B of the research (Lewisham and Croydon NHS governance teams). A flow diagram of the study design is provided in Figs. 1 and 2, and the schedule of enrolment, interventions and assessments is provided in Fig. 3.

The RCT element of the study was suspended and then adapted due to the COVID-19 pandemic and the pausing of research projects. The naturalistic study is currently being undertaken to address issues surrounding the small sample size following the early termination of the RCT. There are currently no plans to resume recruitment in the RCT capacity. Specifically, a naturalistic design is being undertaken, rather than continuing with the original RCT format, due to the suspected difficulties in recruiting enough participants to fulfil both HIIT and TAU arms in the RCT design during covid. New admissions to inpatient wards dropped during the first wave of the pandemic, with an additional 2441 patients discharged from the hospital setting to free up bed capacity for the emergency response and to reduce the risk of infection [50]. This means that there were fewer inpatients with SMI overall, with many of those remaining in inpatient settings experiencing a greater symptom load, a more negative impact on functioning with attendant reduced likelihood and capacity of consenting to research and completing research assessments. The adapted naturalistic design will allow for the collection of feasibility and acceptability data which are the primary outcomes of the pilot study. Moreover, those who are more severely ill are less likely to participate in RCTs of lifestyle interventions [51]; thus, the use of a naturalistic design may provide a greater indication of uptake of HIIT in a clinical capacity.

### Setting

Participants are being recruited from adult inpatient settings operating in one large London mental health National Health Service (NHS) Trust, South London & Maudsley NHS Foundation Trust (SLaM), which provides inpatient and community mental health care for people residing in London boroughs of Lambeth, Southwark, Lewisham and Croydon, along with National Specialist services. The HIIT intervention was conducted, face to face, in the hospital Physiotherapy department for those recruited to the RCT design. The HIIT intervention will be conducted, remotely or face to face, on the inpatient ward for those recruited to the naturalistic design depending on COVID-19 social restrictions.

### Participants

Participants are being recruited from adult inpatient settings operating in SLaM, through adverts and presentations at ward-based community meetings for patients and via clinician referral. Additionally, posters are being displayed onwards with a request that anyone with an interest in taking part should contact the research team. All those who display an interest will be given written information on the study and invited to take part, providing they meet study eligibility criteria.

Individuals are eligible for the study if they meet the following criteria: (1) a service user receiving inpatient care in the participating NHS Trust for a severe mental health condition (including schizophrenia, MDD and bipolar disorder), (2) sufficient command of spoken English, and (3) able to provide written informed consent. Exclusion criteria include (1) a medical condition that impedes exercise (e.g., chronic back pain), (2) an eating disorder (e.g., anorexia nervosa, bulimia nervosa) characterised by excessive exercise, and (3) pregnancy. Additionally, in Part A of the research design eligibility was limited to those aged 18–60 years old.

We aimed to recruit 40 participants to the RCT phase, 20 in the intervention and 20 in the control group over a 1-year period. This number is based on recruiting a big-enough sample to assess the feasibility of the study and on the resources available [52]. We aim to recruit 10 participants to the naturalistic phase of the study. This reduced sample considers COVID-19-related challenges to recruitment, including reduced referrals and admissions to inpatient mental health wards during the pandemic [50].

### Procedure

Participants who satisfy eligibility criteria are being screened using the Physical Activity Readiness Questionnaire (PAR-Q) [53] to determine readiness to exercise. Participants are deemed eligible to exercise according to the PAR-Q if they do not (a) experience chest pain during exercise, (b) have a bone or joint problem (for example, back, knee or hip) or a heart condition that could be made worse by a change in physical activity, or (c) lose balance due to dizziness. In Part A of the study design, participants were asked to give written informed pre-consent before completing the PAR-Q.

Following satisfaction that the participant is ready to exercise according to this screening tool, participants meet with a member of the research team to obtain written informed consent for study participation and complete baseline assessments.

#### RCT design: Part A

Participants completed a sociodemographic questionnaire, mental health assessments, a cognitive test, anthropometric measures, lifestyle assessments and the bicycle YMCA submaximal fitness test [54]. The YMCA fitness test predicts the maximum rate of oxygen consumption (VO2max) during incremental exercise via measuring heart rate changes in response to an increase in resistance. It is an extrapolation method whereby heart rate workload points are gathered and extrapolated to age-predicted maximal heart rate [54]. Mental health assessments, the cognitive test, anthropometric measures, lifestyle assessments and the bicycle YMCA submaximal fitness test were to be repeated at week 6 and week 12 (post-intervention). Participants who were not physically able to complete the YMCA test were excluded from the study due to low baseline fitness and suspected difficulties in exercising at high intensity, this decision was undertaken to minimise the potential occurrence of AEs. Following successful completion of these assessments, the participant was assigned a unique code that was sent to King’s Clinical Trial Unit for randomisation. Participants were randomised in a 1:1 ratio to HIIT or TAU conditions using block randomisation with varying block sizes. Participants and researchers were notified of allocation after completion of all baseline assessments.

Those randomly allocated to the HIIT arm received two, one-to-one, sessions per week for 12 weeks, comprising 24 sessions in total in addition to TAU. HIIT sessions took place in the hospital physiotherapy department and were supervised by a member of the research team, and additional support was provided by a healthcare assistant, nurse or occupational therapist where Section 17 leave conditions stipulated accompany off ward by ward-based staff (Mental Health Act, 1983, [55]).

#### Naturalistic design: Part B

Participants will complete a sociodemographic questionnaire. All participants recruited to the naturalistic study phase will be invited to take part in HIIT, no time limit for participation will be applied. Participants will be able to take part in two HIIT sessions per week for the full duration that the study is running and will not be excluded if they wish to take part at a reduced capacity. HIIT sessions will take place on the ward in which the patient resides. A member of the research team will deliver the session remotely via projector screen with a ward-based member of staff on hand to assist the patient where required or face-to-face (dependent on COVID-19 restrictions).

Additionally, participants will be able to use the bike to conduct self-directed sessions of their own chosen length and intensity either in addition to the supervised HIIT sessions or instead, these sessions will be facilitated by ward staff.

### Intervention

#### High-intensity interval training

Each session takes part on an electronically braked upright stationary bike (Taurus UB9.9 Light Commercial Upright Bike; Cardiostrong BX70i Upright Exercise Bike; Vision Fitness HRT E3200 Upright Cycle). Sessions comprise a 4-min warm-up, five 1-min intervals at 85–95% of maximum heart rate (HRmax) interspersed with 90 s of cycling at 60–70% of HRmax, followed by a 4-min cool-down. The total session duration is 19 min. A 4-min time scale to warm-up and cool-down was deemed an appropriate length based on previous HIIT trials whereby similar length warm-up and cool-down periods have been found to be safe and feasible [28, 42]. The heart rate is recorded throughout each high-intensity interval using handlebar sensors or a chest strap to monitor compliance with target heart rates. The heart rate ranges are achieved by adjusting ergometer resistance and speed. Handlebars only provide an accurate recording of heart rate if held continuously throughout the duration of the high intensity interval; therefore, participants who did not want to continuously hold the handlebar sensors during vigorous exercise were offered the opportunity to wear a chest strap to monitor the heart rate during the RCT phase. The option to utilise a chest strap will not be offered in the naturalistic study phase due to COVID-19-imposed hygiene measures and impracticalities of providing a separate chest strap for each participant that can be washed after each use. External influences on the heart rate, such as co-prescribed beta-blocker medications, will be recorded and presented.

Duration of HIIT is being adapted for those unable to complete the standardised format (e.g., intensity and/or length of sessions reduced), and the intervention incrementally increased as fitness improves. For Part A only, failure to attend sessions for 2 weeks without reason resulted in withdrawal from the study as it was assumed that the participant no longer wanted to take part in the trial. The music is played for the duration of HIIT sessions to maximise motivation.

#### Treatment as usual (Part A Only)

Participants allocated to TAU were instructed to maintain their usual dietary habits, no restrictions were applied to their current level of physical activity.

#### First HIIT session (Part A Only)

Participants completed the Subjective Exercise Experiences Scale (SEES) [56] and self-report measures of appetite and cigarette cravings immediately before and immediately after their first exercise session to determine the acute effects of HIIT on psychological states and cravings. Participants allocated to TAU completed these assessments before and after 19 min of sitting still in quiet, with no distractions such as music or reading material, during the first week of enrolment.

### Outcomes

#### Primary outcomes

Feasibility will be assessed, following completion of both study phases, using multiple primary endpoints: (a) percentage of wards consulted that agree to hosting the project, (b) recruitment numbers and reasons for disinterest, (c) completion rates, (d) attendance to HIIT sessions, (e) adherence to HIIT protocol (number of high-intensity intervals completed per session, peak HR achieved per interval and average HR achieved during each session), and (f) AEs (defined as any untoward medical occurrence in a subject to whom a therapy has been administered including occurrences which are not necessarily caused by or related to that therapy).

Acceptability is assessed at the end of each HIIT session using a single item 10-point Likert scale from 1 (not satisfied) to 10 (most satisfied), and an end of study survey consisting of 12 items for the HIIT group, and 5 items for the TAU group, rated on a 5-point Likert scale (strongly disagree, disagree, neither agree nor disagree, agree and strongly agree) will be employed. This survey was adapted from the acceptability questionnaire originally employed by Chapman et al. [40]. Questionnaire items are shown in Supplementary Tables 1–2, Additional file 1.

Additionally, in Part B we will measure whether patients take part in HIIT sessions or conduct self-directed sessions on the bike. For self-directed sessions, we will ask patients to note whether they follow the HIIT format or conduct a different form of exercise and we will measure satisfaction as above.

#### Secondary outcomes (Part A only)

Secondary outcomes include a range of mental, cognitive and lifestyle outcomes and were collected at 6 weeks and 12 weeks post-randomisation.

##### Mental health status

Symptoms of poor mental health were assessed using the Depression Anxiety Stress Scale (DASS21) [57], the Short Warwick Edinburgh Mental Well-being Scale (SWEMWBS) [58], the Brief Psychiatric Rating Scale (BPRS) [59] and the brief insomnia severity index (ISI) [60]. The DASS21 is a 21-item questionnaire containing 3 subscales (depression, anxiety, stress) of 7 items, with possible scores ranging from 0 to 21. The subscales have high internal reliability in adults across a range of mental illnesses (Cronbach’s alpha of 0.94, 0.87 and 0.91 for depression, anxiety and stress, respectively) [61]. The SWEMWBS is a brief 7-item scale that measures key aspects of psychological functioning, scores range from 7 to 35, validated in people with SMI [58, 62]. The BPRS measures psychiatric symptoms including hallucinations, unusual thought content, emotional withdrawal, depression, anxiety and self-neglect [59] over 18-items, with total scores ranging from 18 to 126, with high inter-rater reliability and validity [63, 64]. The brief ISI is a 7-item questionnaire, with scores ranging from 0 to 28 whereby a score of 15 or above indicates clinical insomnia [60], validated for sleep disorders [60] and used previously in people with SMI [65–67].

##### Cognitive function

The Montreal Cognitive Assessment (MoCA) [68] is a brief screening tool that has high sensitivity and specificity for detecting mild cognitive impairments on a 0–30-point scale whereby a score below 26 indicates cognitive impairment [68].

##### Self-esteem

The Rosenberg Self-Esteem Scale (RSES) [69] measures self-esteem and global self-worth over 10 items, scores range from 0 to 30 [69]. The RSES has adequate internal consistency, test-retest reliability and construct validity in people with SMI [70, 71].

##### Lifestyle measures

The Short-Form International Physical Activity Questionnaire (IPAQ-SF) [72] captures self-reported physical activity and sedentary behaviour over 7 items and has been validated in people with SMI [73, 74] with comparable reliability and criterion validity to that reported in the general population [73, 74]. Motivation to engage in physical activity will be measured via The Behavioural Regulation in Exercise Questionnaire-3 (BREQ-3); it consists of 24 items related to six motivation types (amotivation, external regulation, introjected regulation, identified regulation, integrated regulation and intrinsic regulation) and has been validated in people with schizophrenia [75].

##### Cardiorespiratory fitness

The bicycle YMCA submaximal fitness test measures estimated VO2max [54]. This test provides an inexpensive, quick (15 min to administer) and safe measure of VO2max in comparison with maximal fitness tests which may provide more accurate findings but require the use of expensive or off-putting equipment (such as oxygen masks or mouthpieces) and highly skilled personnel [54]. Moreover, direct measurement requires participations to exercise until exhaustion, which may be challenging for those with lower baseline fitness and those with chronic psychiatric symptoms. The YMCA test has been validated for the prediction of VO2max [54] and has been used to predict VO2max in people with SMI [76, 77]. Additionally, gait speed was measured as the time taken to walk 6 m. Gait speed has been measured using similar estimates in previous studies with people with SMI [78, 79].

##### Anthropometric measures and cigarette intake

BMI, waist circumference (WC), blood pressure and cigarette intake were collected as they provide an indication of cardiometabolic and cardiovascular disease risk [80, 81]. WC was taken from the umbilicus with the participant standing. BMI was calculated based on height and weight. Systolic and diastolic blood pressure were collected via a blood pressure monitor using standardized techniques, two readings were taken at 5-min intervals and the second was used. Participants were asked about their daily cigarette intake.

#### Tertiary outcomes (Part A only)

The SEES assesses psychological states, and it has been validated to assess the effects of exercise on psychological states [56]. This self-report questionnaire contains 12 items, rated on a 7-point Likert scale, subdivided into three subscales to assess immediate feelings of positive well-being, psychological distress and fatigue.

Smokers were asked to rate cigarette craving on a 10-point Likert scale, with higher scores indicating higher levels of cravings. All participants rated appetite craving on a 10-point Likert scale and were additionally asked ‘If you do feel like eating what would it be?’.

### Data management

Each participant is being allocated an anonymised patient identification number (PIN). All information collected will be kept confidential; all identifiable data will be stored in locked filing cabinets. The outcome data is being collected on paper forms and will be transferred to an IBM Statistical Package for the Social Sciences (SPSS) database (SPSS version 27, Chicago, IL, USA). The research ethics committee will be consulted prior to any changes in protocol. The protocol used will be made available on request.

### Safety

During Part A, all AEs and adverse reactions (ARs) (any untoward and unintended response in a subject to a therapy which is related to any duration of therapy administered to that subject), whether serious or not, were to be reported to the ward-based clinical team within 24 h. Serious AEs (SAE), serious ARs (SAR) and unexpected serious ARs (USAR) were to be reported to the participant’s GP. Appropriate action was to be taken including (1) withdrawing the participant from the trial, (2) adapting the exercise regime for the participant (and potentially for other participants) and (3) terminating the trial if necessary.

SAEs, SARs and USARs are defined as any AE, AE or unexpected AR, respectively, that are life-threatening; require hospitalisation or prolongation of existing hospitalisation; or result in significant disability.

During Part B, if an AE or AR occurs during exercise, the session will be stopped and a physical health check will be carried out if required. The clinical team will be consulted. Depending on the severity of the event, the exercise regime may be adapted for that patient or it may be decided that they are not able to exercise at their current physical health status.

### Data analysis

Acceptability and feasibility outcomes will be reported post-randomisation and summarised by treatment arm (HIIT and TAU) and study phase (RCT and naturalistic phase). Descriptive statistics including percentages and raw figures will be used to assess feasibility outcomes and acceptability survey responses. All AEs will be listed. Satisfaction scores (a measure of acceptability) will be collated to form a mean satisfaction rating.

Data analysis, for secondary and tertiary measures, will be simplified to account for small sample size owing to suspension of the RCT in line with COVID-19-inflicted restrictions. Data analysis plan suggested a formal statistical analysis whereby secondary outcome variables measured at the intervention time points (6 and 12 weeks) were to feature as dependent variables with, treatment arm (HIIT or TAU), time (6 or 12 weeks), baseline BPRS, baseline fitness and baseline anthropometric measurements included as covariates.

In lieu of the above plan, to evaluate effectiveness of the intervention in line with our secondary aim, changes pre- and post-intervention in mental and physical parameters will be assessed, and Cohen’s *d* and confidence intervals will be calculated to determine between-group effect size for mean change. This secondary analysis will be based on the intention-to-treat sample.

Similarly, for the tertiary outcomes, changes pre- and post-intervention in psychological wellbeing, psychological distress, fatigue and appetite and cigarette cravings following a single bout of HIIT will be assessed, and Cohen’s *d* and confidence intervals will be calculated to determine between-group effect size for mean change. As above, this analysis will be based on the intention-to-treat sample. An additional analysis will be conducted with those participants who adhered to the HIIT protocol (completed the full 19-min HIIT session and who reached the target heart rate).

Data analysis will be conducted using Statistical Package for the Social Sciences (SPSS) version 27 (Chicago, IL, USA).

### Reimbursements

Participants will not receive payment, although complementary reusable water bottles and gym tops will be provided.

In Part A of the study, participants who were discharged from the inpatient setting over the course of the trial were invited to return to the hospital to resume HIIT sessions, subject to COVID-19 social restrictions, and associated travel costs were reimbursed. In Part B of the study, participants who are discharged from the inpatient setting will not be invited to the hospital to resume HIIT sessions due to insurance limitations, COVID-19 social restrictions and simplistic nature of the study.

### Follow-up

Participants who took in part A of the pilot study were invited to take part in individual semi-structured follow-up qualitative interviews. We hoped to conduct each qualitative interview within 3 weeks of each participant finishing the HIIT pilot study/ within 3 weeks of drop-out. We aim to find out (1) how participants experienced the intervention, (2) if there are any parts of the intervention that could be improved and (3) what factors influenced people in completing/not completing the intervention.

Qualitative interviews took place via telephone or video call. Each qualitative interview begun with the issuing of consent forms. Then, a series of simple questions about the positive aspects and weaknesses of the intervention were asked, and participants answered verbally. Overall, each individual interview was scheduled to take 30–60 min.

Material gained from the follow-up will be analysed using thematic analysis [82]. The themes generated will address the barriers and facilitators and perceived enjoyment of the HIIT intervention and areas for improvement for future work. As such data generated will seek to complement the acceptability and feasibility data gathered from the primary trial endpoints. We sought to offer an interview to all patients recruited to Part A of the study.

### Criteria to indicate that a future effectiveness trial is feasible

At present, no large-scale study is planned; however, feasibility outcomes will provide an indication as to whether a future effectiveness trial is warranted [48]. Criteria that indicate that a future effectiveness trial is feasible include (1) a majority of wards agreeing to hosting the project, (2) meeting stated recruitment (40 participants to the RCT and 10 participants to the naturalistic study phase), (3) comparable completion rates and adherence to other exercise interventions carried out in people with SMI (drop-out rates of previous exercise interventions in people with SMI has averaged 24–32.5% and adherence to scheduled sessions has averaged 55–94% [41, 42, 83], (4) lack of serious exercise-related AEs and (5) participants expressing that the conduct of the intervention was acceptable.

### Patient and public involvement

The research design and participant facing documents were reviewed by a team with experience of mental health problems and their carers who have been specially trained to advise on research proposals and documentation through the Young Person’s Mental Health Advisory Group (YPMHAG) and the Feasibility and Acceptability Support Team for Researchers (FAST-R): two separate free, confidential services in England provided by the National Institute for Health Research Maudsley Biomedical Research Centre via King’s College London and South London and Maudsley NHS Foundation Trust. Moreover, an ex-service user was consulted in the design process and with knowledge in mental health disorders and exercise, both from personal experience and undergraduate study, was consulted in the design process.

Focus groups were conducted with inpatients with SMI, carers and clinical staff to investigate perspectives on implementing HIIT interventions for service users in inpatient settings, including perceived barriers and enablers (discussed in background section above) [47]. Practical and safety considerations were noted and methods to increase patient motivation were sought and incorporated into the research design.

## Discussion

This pilot study aims to evaluate the feasibility and acceptability of an exercise intervention for inpatients with SMI. To the best of our knowledge, no HIIT interventions have been developed and tested in the NHS. This study will allow us to understand the feasibility of deploying the HIIT intervention itself and the use of the primary, secondary and tertiary outcome measures. Upon completion of the study, we will have a detailed overview of whether the delivery and planned evaluation approach are feasible and acceptable to deliver as part of a multi-centre large-scale definitive trial; namely whether participants agree to take part, continue to attend HIIT sessions and assessments and adhere to the HIIT protocol. The design of the study, with both RCT and naturalistic study design elements, will allow us to determine feasibility and acceptability of HIIT research when delivered in both research and clinical/naturalistic settings. Both phases of the study will address the feasibility and acceptability of delivering HIIT, while the RCT element will offer an indication to whether mental and physical health measures can be obtained providing an indication as to whether a large scale RCT of HIIT is the best design to determine its clinical effectiveness. With this said, the design of the study has various limitations. Suspension of the RCT study phase due to COVID-19 will limit power to detect changes in secondary and tertiary outcomes, and these outcomes will not be measured during the naturalistic study phase. Moreover, recruitment is limited to one NHS trust (although participants will be recruited from three sites), and reliability of measures of adherence to target heart rate will be limited in the naturalistic study phase due to inability to use chest straps for monitoring heart rate.

The physical health of people with SMI remains a high priority, and exercise has been identified as a modifiable lifestyle barrier that has the potential to reverse cardiovascular and cardiometabolic disease risk and reduce associated premature mortality. The National Institute for Health and Care Excellence (NICE) guidelines recommend that interventions be offered to support people with SMI to be more active [84]. HIIT is a time-efficient intervention that has yielded benefits for physical and mental health in other settings and populations [28]. If HIIT is found to be feasible and acceptable in inpatients with SMI, there would be scope for large-scale work and the potential implementation of this intervention in inpatient mental health facilities.

### Trial status

The RCT element of the study was suspended due to the COVID-19 pandemic and then adapted to a naturalistic study design which is ongoing, and recruitment is open.

## Supplementary Information

**Additional file 1 **: **Supplementary Table 1**. HIIT Survey for trial endpoint HIIT arm. **Supplementary Table 2**. HIIT Survey for trial endpoint TAU arm.

## Data Availability

The datasets used during the current study may be available from the corresponding author on reasonable request.

## Abbreviations

AE: Adverse events
AR: Adverse reaction
BPRS: The Brief Psychiatric Rating Scale
BREQ-3: The Behavioural Regulation in Exercise Questionnaire-3
DASS21: Depression Anxiety Stress Scale
EPA: European Psychiatric Association
FAST-R: The Feasibility and Acceptability Support Team for Researchers
HIIT: High-intensity interval training
HRmax: Maximum heart rate
IPAQ-SF: The Short-Form International Physical Activity Questionnaire
ISI: The brief insomnia severity index
MDD: Major depressive disorder
MICT: Moderate-intensity continuous training
MoCA: Montreal Cognitive Assessment
NICE: The National Institute for Health and Care Excellence
NHS: National Health Service
PAR-Q: Physical Activity Readiness Questionnaire
PIN: Patient identification number
RCT: Randomised controlled trial
RSES: The Rosenberg Self-Esteem Scale
SAE: Serious adverse events
SAR: Serious adverse reaction
SEES: Subjective Exercise Experiences Scale
SLaM: South London & Maudsley NHS Foundation Trust
SMI: Severe mental illnesses
SPSS: Statistical Package for the Social Sciences
SWEMWBS: Short Warwick Edinburgh Mental Well-being Scale
TAU: Treatment as usual
USAR: Unexpected serious ARs
WC: Waist circumference
YPMHAG: The Young Person’s Mental Health Advisory Group
