# Supplementary material for: Study protocol for a pilot high-intensity interval training intervention in inpatient mental health settings: a two-part study using a randomised controlled trial and naturalistic study design
Source: Pilot Feasibility Stud. 2021 Nov 8;7:198. doi: 10.1186/s40814-021-00937-6 (PMC8573298; doi:10.1186/s40814-021-00937-6)
Supplement: Supplementary file 1 — Additional file 1 : Supplementary Table 1. HIIT Survey for trial endpoint HIIT arm. Supplementary Table 2. HIIT Survey for trial endpoint TAU arm. [file 40814_2021_937_MOESM1_ESM.docx]

**Supplementary Table 1, Additional File 1- HIIT Survey for trial endpoint HIIT arm**

Please tick as appropriate.

|  | **Questionnaire Item** | **Strongly Agree** | **Agree** | **Neutral** | **Disagree** | **Strongly Disagree** |
| --- | --- | --- | --- | --- | --- | --- |
| 1. | I feel satisfied with the condition I was allocated to |  |  |  |  |  |
| 2. | I would have preferred to be in the other condition |  |  |  |  |  |
| 3. | I found the exercise enjoyable |  |  |  |  |  |
| 4. | I found the exercise boring |  |  |  |  |  |
| 5. | I found the exercise too hard |  |  |  |  |  |
| 6. | I enjoyed individual exercise sessions |  |  |  |  |  |
| 7. | I would have preferred group-based exercise |  |  |  |  |  |
| 8. | I looked forward to the exercise sessions |  |  |  |  |  |
| 9. | The trial has motivated me to be more active |  |  |  |  |  |
| 10. | I will engage in exercise now the trial has finished |  |  |  |  |  |
| 11. | I am motivated to engage in HIIT now the trial has finished |  |  |  |  |  |
| 12. | I feel confident to engage in HIIT exercise without supervision |  |  |  |  |  |

**Supplementary Table 2, Additional File 1- HIIT Survey for trial endpoint TAU arm**

Please tick as appropriate.

|  | **Questionnaire Item** | **Strongly Agree** | **Agree** | **Neutral** | **Disagree** | **Strongly Disagree** |
| --- | --- | --- | --- | --- | --- | --- |
| 1. | I feel satisfied with the condition I was allocated to |  |  |  |  |  |
| 2. | I would have preferred to be in the other condition |  |  |  |  |  |
| 3. | The trial has motivated me to be more active |  |  |  |  |  |
| 4. | I will engage in exercise now the trial has finished |  |  |  |  |  |
| 5. | I am motivated to engage in HIIT now the trial has finished |  |  |  |  |  |
